# Timing Rhythms: Perceived Duration Increases with a Predictable Temporal Structure of Short Interval Fillers

**DOI:** 10.1371/journal.pone.0141018

**Published:** 2015-10-16

**Authors:** Ninja K. Horr, Massimiliano Di Luca

**Affiliations:** Centre for Computational Neuroscience and Cognitive Robotics, Department of Psychology, University of Birmingham, Edgbaston, Birmingham, B15 2TT, United Kingdom; Durham University, UNITED KINGDOM

## Abstract

Variations in the temporal structure of an interval can lead to remarkable differences in perceived duration. For example, it has previously been shown that isochronous intervals, that is, intervals filled with temporally regular stimuli, are perceived to last longer than intervals left empty or filled with randomly timed stimuli. Characterizing the extent of such distortions is crucial to understanding how duration perception works. One account to explain effects of temporal structure is a non-linear accumulator-counter mechanism reset at the beginning of every subinterval. An alternative explanation based on entrainment to regular stimulation posits that the neural response to each filler stimulus in an isochronous sequence is amplified and a higher neural response may lead to an overestimation of duration. If entrainment is the key that generates response amplification and the distortions in perceived duration, then any form of predictability in the temporal structure of interval fillers should lead to the perception of an interval that lasts longer than a randomly filled one. The present experiments confirm that intervals filled with fully predictable rhythmically grouped stimuli lead to longer perceived duration than anisochronous intervals. No general over- or underestimation is registered for rhythmically grouped compared to isochronous intervals. However, we find that the number of stimuli in each group composing the rhythm also influences perceived duration. Implications of these findings for a non-linear clock model as well as a neural response magnitude account of perceived duration are discussed.

## Introduction

Perceived duration of an interval is influenced by interval filling. A well-known and consistent effect demonstrating this influence is the filled-duration illusion [1–4]: Intervals demarcated by a beginning and an end marker are perceived to last longer if they contain a number of short filler stimuli (filled intervals) rather than if there is no stimulation between the two markers (empty intervals). Recently it has been shown that not only the number and duration of interval fillers make a difference in perceived duration [2,5], but also their temporal structure plays a role [6–8]. Here, we intend to further explore the role of temporal structure on perceived duration.

Effects of temporal structure on perceived duration could be explained in the framework of an accumulator-counter mechanism by hypothesizing a non-linear accumulator that is resetting at the onset of every stimulus delimiting a subinterval [2,7]. The overall duration is then the sum of each accumulated subinterval. Such a clock model with a logarithmic accumulator adheres to the empirical finding of a decrease in perceived duration with higher filler anisochrony, i.e. randomness [6], as whatever is added to the physical duration of one subinterval will be perceptually less than the same physical duration being subtracted from the other subintervals. The logarithmic clock model also predicts the finding that a higher number of filler stimuli increases the perceived duration difference between temporally regular compared to temporally irregular intervals (see [6] for mathematical model).

An alternative explanation for distortions due to temporal structure is based on the relation between perceived duration and neural response magnitude. It has been proposed [9,10] that a higher neural response to a stimulus leads to a longer perceived duration, for example due to a stronger representation in memory (memory trace). Recent studies have provided experimental evidence that neural response magnitude can account for perceived duration, both with monkey single cell recordings techniques [11–12] and with human magnetoencephalography methodologies [13–14]. In this context, the increase in perceived duration with more filler stimuli can be explained by a higher cumulative neural response [2,5]. On the other hand, changes in perceived duration due only to differences in temporal structure require further explanation. An interesting phenomenon that stems from temporal regularity is the entrainment of neural activity [15–18]. Exposure to isochronous stimulation leads to modification of the phase of neural oscillations so that the isochronous stimuli arrive at the peak of neural oscillations. It has further been shown that stimulus processing is modulated by the phase of neural oscillations, that is, the point in time at which a stimulus arrives determines whether the elicited signal is amplified or attenuated [19,20]. Consistent with this idea, Lakatos and colleagues [16] suggested that neural entrainment guides attentional selection, so that response gain is higher for stimuli arriving in phase with the neural oscillation. Isochronous intervals would therefore elicit higher neural responses than equivalent anisochronous intervals because each stimulus would arrive at the point of highest response amplification. For anisochronous sequences, instead, each stimulus would arrive at a random phase of the oscillation, so that there is equal probability of amplification and attenuation, that evens out overall modulation due to oscillatory phase. Linking this back to a response magnitude account of perceived duration, an overestimation of isochronous as compared to random stimulation due to entrainment is what would be expected.

In the present line of experiments, we test whether and how rhythmic structures in the stimuli filling an interval influence perceived duration. Investigating such changes in perceived duration allows us to better understand the role of temporal structure on perceived duration and to explore to what extent it is in line with the two accounts of duration distortion (non-linear clock and entrainment). The predictions of a non-linear clock model can be calculated for stimuli of any kind of structure, including the rhythmic ones we use here. The reason for using a rhythmic structure is that the timing of each filler stimulus is fully predictable just after listening to the first rhythmic group. In such a case, neural entrainment should happen in the same way as for isochronous stimulation and rhythmic, just like isochronous intervals, should be perceived as longer than random intervals. We further explore whether there is a difference in perceived duration between isochrony and different types of rhythms.

A two-interval forced-choice task was used to compare the perceived duration of different rhythmic against random or isochronous intervals. In every trial, participants reported which of two intervals was the longer one. To exclude possible response biases the order of the two compared interval types as well as their duration (standard 1000ms or comparison of several durations up to ±500ms) was counterbalanced and randomized. In Experiment 1 we presented participants with one rhythmic and one random interval. According to the non-linear clock model account, which predicts decreased duration with increased randomness in the interval, as well as the neural magnitude account, proposing increased duration due to entrainment, we hypothesized that predictable rhythmic intervals will be perceived to last longer than their random counterparts. In Experiment 2 the same rhythmic intervals were compared against isochronous sequences to determine whether duration estimates differed due to rhythm type despite complete predictability of stimulus timing for both intervals.

## Experiment 1

To test the hypothesis that predictability in a stimulus sequence generally leads to an overestimation of perceived duration and to what extent this effect may be related to the rhythmic structure of the interval, we asked participants to compare intervals of different rhythm types to randomly timed intervals.

### Material and Methods

#### Participants

Twenty-four volunteers (15 female, 20.5±2.7 years) that reported having normal hearing participated in the experiment for course credits or a payment of 7 GBP. Written consent was obtained from each participant. Experimental data collection and storage followed the ethical guidelines of the Declaration of Helsinki (2012) and was approved by the Science, Technology, Engineering & Mathematics Ethical Review Committee of the University of Birmingham.

#### Judgment Intervals

Participants performed duration judgments on auditory intervals filled with a varying number of 1000Hz tones at 70 dB SPL which lasted 10ms with 1ms on and off ramp. In every trial, two intervals (one rhythmic and one randomly timed) with the same number of stimuli were presented in succession. Random intervals were created by randomly moving the onset of individual filler tones (except the beginning and end tone) in an originally isochronous sequence within a range of half of the isochronous interstimulus interval. For rhythmic intervals, groups of tones were presented with a fixed interstimulus interval and the tone between every two groups was omitted. In the following we will refer to the rhythms according to the number of stimuli in each of the rhythmic groups (i.e., a rhythm with *n* stimuli in each group is called group-of-n rhythm). Four rhythm conditions were defined according to the number of stimuli within a group, that is, group-of-2, group-of-3, group-of-4 and group-of-5 rhythms. To determine whether there is an influence not only of the number of stimuli within a group, but also of the number of groups per sequence, group-of-2 rhythms could consist of three, four or five groups per interval. For the other three types of rhythms there were three groups per interval.

#### Experimental Design

**Fig 1:**
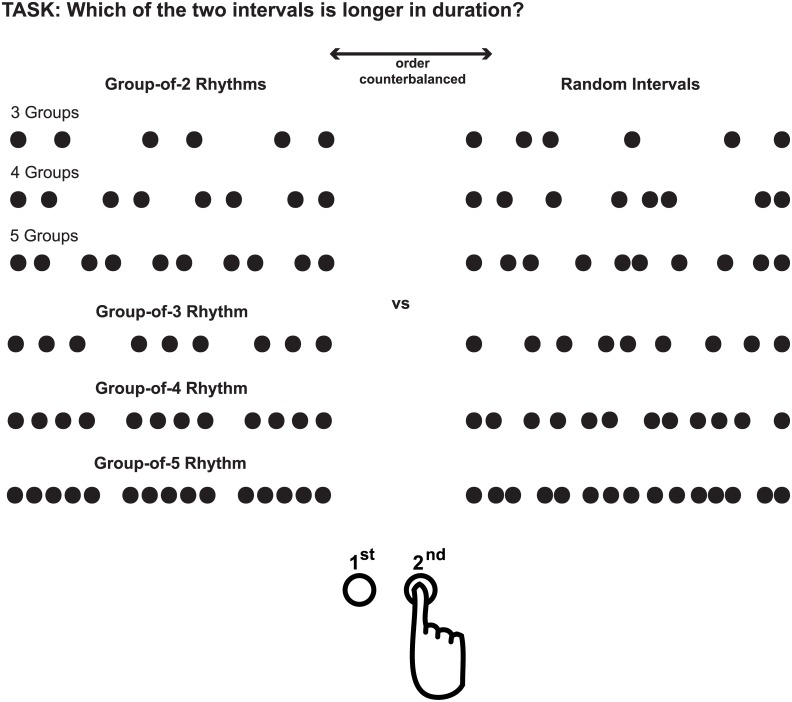
Experimental Task in Experiment 1. Intervals compared in Experiment 1 displayed for two intervals of equal duration. Each rhythmic interval was compared to a random interval. The number of tones was always equal for the two intervals compared and their order was counterbalanced.

Duration judgments were obtained in a two-interval forced-choice task. In every trial, participants pressed a button corresponding to which of two intervals appeared to last longer, the left button for the first one or the right button for the second one. One of the two intervals was always rhythmic, the other one random. One interval was always 1000ms long, while the other interval had a duration of 500ms, 700ms, 850ms, 1000ms, 1150ms, 1300ms or 1500ms. Varying durations of an interval solely changed the frequency of filler tones, while the number of fillers as well as their relative temporal relationships stayed intact. The different rhythmic patterns were presented blocked with the sequence of blocks randomized between participants. Fig 1 schematically displays the task and the temporal structure of intervals compared.

In total, participants made 336 duration discrimination judgments in 6 blocks of 56 trials each. The order of presentation of the rhythmic and random interval as well as the order of the 1000ms interval and the variable duration interval was randomized and counterbalanced within each block. That is, in a quarter of the trials each the rhythmic interval was (a) 1000ms long and preceded by a varying random interval, (b) 1000ms long and followed by a varying random interval, (c) varying in duration and preceded by a 1000ms random interval and (d) varying in duration and followed by a 1000ms random interval. The sequence of trials was differently randomized for each participant. The experimental session lasted about 1 hour.

Participants’ individual response proportions were assessed in relation to the physical duration difference between the rhythmic and the random interval. With 56 trials per block and 7 possible durations compared to 1000ms, there were 8 repetitions at every duration difference. The point of subjective equality (PSE) and the just noticeable difference (JND) were estimated using the Spearman-Kärber-Method as the first and second moment of the distribution underlying the raw data obtained from each participant [21]. With p_i_ being response proportions and s_i_ being the 7 duration differences between the rhythmic and the random interval presented at each trial, we define s_0_ = -1350ms and and s_8_ = 1350ms and we assume p_0_ = 0 and p_8_ = 1. PSE and JND can then be derived analytically as such:

$$PSE=\overset{8}{\underset{i=1}{\sum^{\text{​}}}}\frac{p_{i}-p_{i-1}}{2}(s_{i}-s_{i-1})$$

$$JND=\sqrt{\overset{8}{\underset{i=1}{\sum^{\text{​}}}}\frac{p_{i}-p_{i-1}}{2}{\left((s_{i}-s_{i-1})-PSE\right)}^{2}}$$

## Results and Discussion

**Fig 2:**
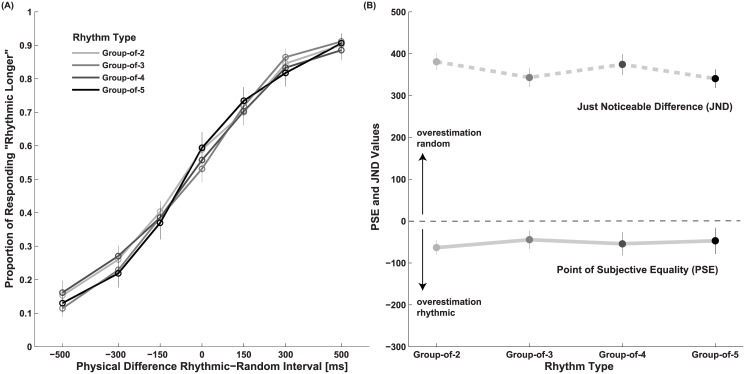
Results of Experiment 1. (A) Proportions of responses indicating the rhythmic interval to be longer than the random interval as a function of physical duration difference. (B) Point of subjective equality (PSE) and just noticeable difference (JND) calculated from response proportions with the Spearman-Kärber method. Error bars are S.E.M.

Response Proportions, PSE and JND values separated by rhythm condition are shown in Fig 2. As there is no significant difference between the PSE and JND for the three group-of-2 rhythms with different number of groups (one-way r.m. ANOVA on PSE: *F*(2,46) = 0.37, *p* = 0.69, *η*_*p*_ *² = 0*.*02*; on JND *F*(2,46) = 0.57, *p* = 0.56, *η*_*p*_ *² = 0*.*02*), the results for the group-of-2 rhythms are presented together. The PSE averaged across the four rhythmic conditions is significantly lower than zero (-60±18ms, t-test against 0, two-tailed: *t(23)* = -3.1, *p* = 0.006, *d* = 0.62), indicating that rhythmic intervals are perceived as longer than anisochronous intervals. The duration required for an anisochronous stimulus to match a rhythmic one does not differ for rhythms composed of groups of different number (one-way r.m. ANOVA on PSE, *F*(3,69) = 0.24, *p* = 0.87, *η*_*p*_ *² = 0*.*01*). The overall JND indicates that people were able to discriminate within the given range of 500ms duration difference (378±18ms). JND does not significantly vary across conditions (one-way r.m. ANOVA on JND, *F*(3,69) = 1.93, *p* = 0.13, *η*_*p*_ *² = 0*.*08*). Overall, the data highlights a general underestimation of the duration of random intervals compared to rhythmic intervals.

## Experiment 2

To test whether the change of perceived duration due to rhythmic structure as compared to random filling is solely due to the predictability of stimulus timing, we asked participants to compare the duration of intervals composed of two fully predictable sequences of stimuli, one rhythmic and one isochronous.

### Material and Methods

**Fig 3:**
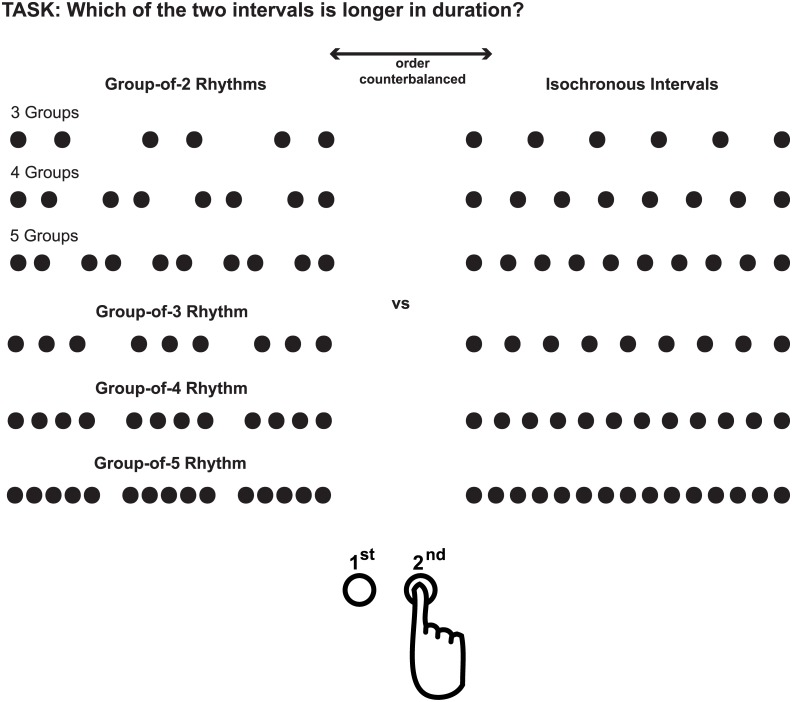
Experimental Task in Experiment 2. Intervals compared in Experiment 2 displayed for two intervals of equal duration. Each rhythmic interval was compared to an isochronous interval. The number of tones was always equal for the two intervals compared and their order was counterbalanced.

Twenty-four new volunteers (12 female, 21.3±2.4years) participated in Experiment 2. Experimental procedure and ethical guidelines were similar as in Experiment 1. Experiment 2 differed from Experiment 1 only in the replacement of random intervals by isochronous intervals. That is, in every trial one rhythmic interval was compared to one isochronous interval. Again the experiment consisted of 6 blocks defined by the six rhythmic patterns. Task and interval structures are displayed in Fig 3. Participants made 336 duration discrimination judgments, with 56 trials per block, that is, 56 trials comparing a specific type of rhythm to an isochronous interval with the same number of stimuli.

### Results and Discussion

**Fig 4:**
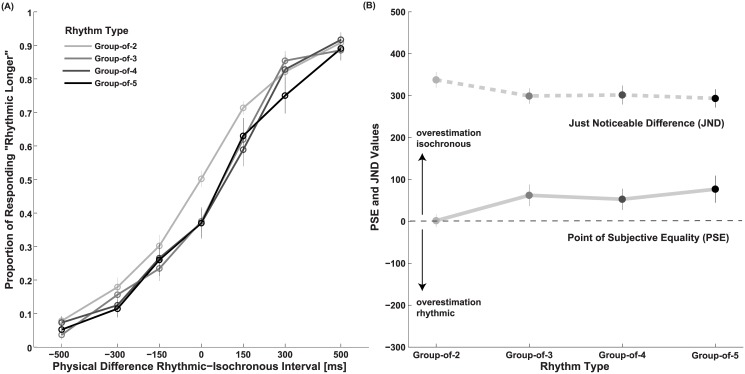
Results of Experiment 2. (A) Proportions of responses indicating the rhythmic interval to be longer than the isochronous interval as a function of physical duration difference. (B) Point of subjective equality (PSE) and just noticeable difference (JND) calculated from response proportions with the Spearman-Kärber method. Error bars are S.E.M.

Fig 4 shows response proportions, PSE, and JND values. As in Experiment 1, the three group-of-2 patterns did not differ in terms of PSE or JND and were grouped together (one-way r.m. ANOVA on PSE: F(2,46) = 0.11, p = 0.90, *η*_*p*_ *² = 0*.*08*; on JND: F(2,46) = 2.07, p = 0.14, *η*_*p*_ *² = 0*.*08*). The PSE averaged across the four rhythmic conditions shows a tendency of rhythmic intervals to be perceived as shorter than isochronous intervals, however this underestimation is not statistically significant (33±20ms, t-test against 0, two-tailed: *t*(23) = 1.9, *p* = 0.07, *d* = 0.39). The duration required for an isochronous interval to perceptually match a rhythmic interval changes depending on the rhythm condition (one-way r.m. ANOVA on PSE: *F*(3,69) = 3.3, *p* = 0.027, *η*_*p*_ *²* = 0.13). For each individual rhythm condition the perceived duration of rhythms is not significantly different to the isochronous intervals (t-test of PSE against 0, two-tailed, Bonferroni-corrected: *t*(23) = 0.10, *p*>1, *d* = 0.02, group-of-3: *t*(23) = 2.43, *p* = 0.096, *d* = 0.49; group-of4: *t*(23) = 2.08, *p* = 0.192, *d* = 0.43, group-of-5: *t*(23) = 2.4, *p* = 0.112, *d* = 0.48). Visual inspection suggests that the main effect of rhythm condition on PSE is carried by the difference between group-of-2 rhythms and rhythms with more than two stimuli per group, but these differences cannot be confirmed statistically (paired sample t-test on PSE, two-tailed, Bonferroni-corrected between group-of-2 and: group-of-3 *t*(23) = 2.51, p = 0.112, d = 0.51; group-of-4 *t*(23) = 2.56, p = 0.054, *d* = 0.52; group-of-5 *t*(23) = 2.38, p = 0.078, *d* = 0.48). The average JND is similar to the one obtained in Experiment 1 (330±20ms) and does not vary across conditions (one-way r.m. ANOVA: *F*(3,69) = 2.00, *p* = 0.074, *η*_*p*_ *²* = 0.10). In sum, we do not observe a statistically significant difference in perceived duration between isochronous and fully predictable rhythmic intervals, but we register a change in perceived duration depending on the number of stimuli in the groups of the rhythmic interval.

## General Discussion

The present line of experiments were set out to investigate how the rhythmic structure of interval fillers influences perceived duration. More specifically, we tested whether the observed overestimation of isochronous as compared to random intervals [8] can be due to the predictable temporal pattern of isochronous intervals. We further explored whether different temporal patterns, even if fully predictable, lead to differential distortions of perceived duration. In Experiment 1 we found that rhythmic intervals are perceived to last longer than random intervals. Experiment 2 did not reveal a difference in perceived duration between isochronous and rhythmic intervals.

The overestimation of isochronous compared to random intervals can be accounted for by both a non-linear clock model with a logarithmic accumulator reset at the beginning of every subinterval and by a neural response magnitude account assuming that filler stimuli which arrive at a predictable point in time lead to increased responses due to entrainment [8]. The predictions of the two models for isochronous as compared to rhythmic intervals, instead, are not immediately evident. In the following we will take a closer look at those and discuss to what extent these two models fit the present data.

### Non-linear Clock Model

It has been shown that a logarithmic accumulation of perceived duration in an interval clock framework could explain the overestimation of perceived duration due to isochrony [3,7]. Furthermore, such a non-linear accumulation would predict the observed increase of this effect with increasing anisochrony and with increasing sequence length [8]. Would a non-linear clock model also predict an overestimation of rhythmic sequences as compared to anisochrony? What would it say about the comparison of isochrony and rhythms?

To simulate PSE values from the non-linear clock model, the physical duration *T* ^*1*^ that is needed for an interval to be perceived of equal duration as another interval *T* ^*2*^ can be expressed by

$$\psi \left(T^{1}\right)=\psi \left(T^{2}\right)$$

Where *ψ* represents the psychometric function that relates physical to perceived duration. The non-linear clock model assumes that: (1) the clock is reset at every filler tone demarcating the beginning of a new subinterval [22], (2) the complete interval duration is obtained by summing up the perceived durations of the subintervals *D*, that is $\psi \left(\text{T}\right)=\sum_{s=1}^{N}\psi \prime ({\text{D}}_{s})$, and (3) the relationship between the physical and the perceived duration of the subintervals is logarithmic [3,7] *ψ*′(*D*) = log(*D*).

This leads to:

$$\sum_{S=1}^{N}\text{log}(D_{s}^{1})=\;\sum_{S=1}^{N}\text{log}(D_{s}^{2})$$

which by applying the sum rule of the logarithm simplifies to $\prod_{S=1}^{N}D_{s}^{1}=\prod_{s=1}^{N}D_{s}^{2}$

**Fig 5:**
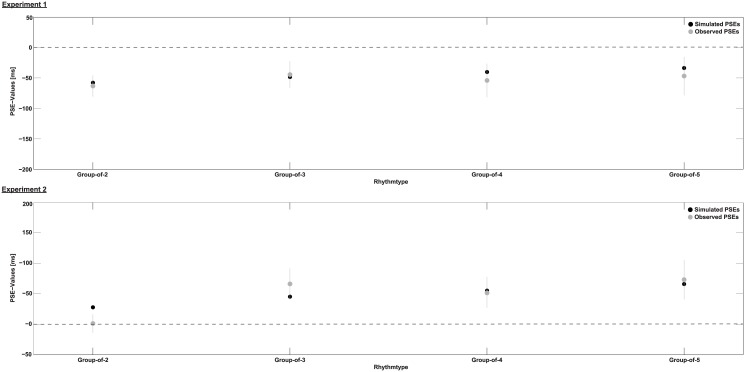
Simulation of PSE values according to a nonlinear clock model. Observed and simulated PSE values. In Experiment 1 simulated PSE values are the mean over 1000 repetitions. In Experiment 2 there is only one simulated PSE value due to the deterministic temporal distribution. The x-axes represent the different comparison conditions as they were in the experiment. Error bars for observed PSE values are S.E.M.

The PSE value is obtained by setting either $\sum_{s=1}^{N}D_{s}^{1}=1\text{s}$ and thus $\sum_{S=1}^{N}D_{s}^{2}=\text{PSE}$ or vice versa. Fig 5 shows the PSE values obtained for the different rhythms. In Experiment 1 we simulated the anisochronous intervals by drawing the mean over 1000 random samples. In Experiment 2 sampling is not necessary as the timing of the filler stimuli is completely determined. In general, the simulated PSE values from Experiment 1 and 2 indicate an underestimation of random and an overestimation of isochronous intervals as compared to rhythmic intervals. They further show a general tendency of a decrease in perceived duration with rhythmic groups containing more stimuli. The results of the simulation have a pattern similar to the observed data. There is no significant difference between observed and simulated PSE values between any of the groups in Experiment 1 (Bonferroni-corrected one-sample t-tests against simulated value, p>1) and Experiment 2 (Bonferroni-corrected one-sample t-test against simulated value for group-of-2, t(23) = 1.9, p = 0.28, all others p>1). The model prediction of an ovestimation of perceived duration for rhythmic intervals as compared to anisochronous intervals is in line with the results of Experiment 1. However, the predicted overestimation of duration for isochronous over rhythmic intervals is not confirmed by the results of Experiment 2. Experiment 1 shows no differential distortions between rhythmic conditions, whereas the results of Experiment 2 are in line with the predictions that rhythms composed of groups with fewer stimuli should be perceived to last longer.

### Entrainment/Neural Response Magnitude Model

The overestimation of rhythmic intervals as compared to random ones observed in Experiment 1 is predicted from a model where the increase of neural response magnitudes due to entrainment translates to an increase in perceived duration [6,15–18]. If we assume that any fully predictable rhythm would generate a similar amount of entrainment, then we should not expect differences in perceived duration between isochronous and rhythmic intervals and all rhythmic groupings should be perceived as having similar duration. The results of Experiment 1 do not highlight a change in perceived duration as a function of group size. Moreover, the results of Experiment 2 show the expected similar perceived duration of rhythmic and isochronous stimuli, but they also highlight an unexplained change in perceived duration as a function of the number of stimuli composing the rhythmic groups. This difference makes it worth thinking about whether and how a model based on entrainment plus neural response magnitude could explain differences between different rhythmic groupings.

A possibly crucial difference between grouping conditions may lie in the number of stimuli that it takes to be able to make predictions on the arrival of a future stimulus. In an isochronous sequence, the inter-stimulus-interval (ISI) between only two stimuli is sufficient to predict the arrival of every other stimulus in the sequence. To make the same prediction in a rhythmic interval, there are several pieces of information required, that is, (a) the ISI between two stimuli, (b) the number of stimuli in a group, and (c) the ISI between two groups of stimuli. Therefore, the observer will necessarily have to wait for the onset of the first stimulus in the second group of stimuli to be able to predict the timing of all of the following stimuli. To sum up, in order to accurately predict all following stimuli, it takes two stimuli in the isochronous sequence, three stimuli in a group-of-2, four stimuli in group-of-3, five stimuli in a group-of-4 and six stimuli in a group-of-5 rhythm. As prediction is delayed, entrainment and thus amplification of neural response in rhythmic intervals may start later, consequently decreasing the overall neural response magnitude and leading to a shorter perceived duration. The predictions of this account would be qualitatively in line with the predictions of a non-linear clock model, namely, a linear decrease of perceived duration with increased number of stimuli per group. Future studies with a wide range of stimuli per group and a direct comparison between different rhythm types are necessary to test whether such predictions hold. An alternative approach to explain distortions between different rhythmic intervals in the entrainment/neural response magnitude framework may be chunking mechanisms that gear phase locking towards the rhythmic groups rather than the individual tones [23–24].

## Conclusions

Previous research has shown that isochronous intervals are overestimated as compared to anisochronous intervals [6]. The present experiments demonstrate that fully predictable rhythmic structures influence perceived duration in the same way as isochrony. This type of temporal distortion suggests that a temporal structure that allows the prediction of stimulus timing increases the perceived duration of intervals.

Both non-linear clock models and the proposal of a connection between perceived duration and entrainment strength due to neural response magnitudes could explain the observed overestimation of isochronous as well as rhythmic intervals compared to random interval filler spacing. The interval clock model predicts a decrease of perceived duration with rhythms composed of more stimuli. The predictions of the magnitude model depend on whether we assume equal or different entrainment strengths for different rhythmic structures. Further research is needed to put additional constraints on a model explaining perceived duration distortions due to temporal structure. Such research should use broader ranges of grouping numbers and directly compare different interval types to determine whether the overestimation of predictable intervals is equivalent for all rhythms including isochrony and, if not, to disentangle general patterns of distortions between such interval types.

## Supporting Information

S1 Table Proportion of Responses. Response Proportions from both Experiments. Individual subjects proportions of responding „rhythmic longer”for every duration difference between rhythmic and random/isochronous interval, separated by condition.
(XLSX)

S2 Table PSE and JND. PSE and JND values from both Experiments. Individual subjects PSE and JND values obtained from response proportions via the Spearman-Kärber-Method, separated by conditions.
(XLSX)
